# Fidgeting Increases Pupil Diameter During Auditory Processing in Young Healthy Adults

**DOI:** 10.3390/brainsci16020127

**Published:** 2026-01-24

**Authors:** Satoko Kataoka, Hideki Miyaguchi, Chinami Ishizuki, Hiroshi Fukuda, Masanori Yasunaga, Hikari Kirimoto

**Affiliations:** 1 Faculty of Health Sciences, University of Kochi Health Sciences, Kochi 781-5103, Japan; kataoka@ko-ken-k3.ac.jp (S.K.); hmiya@ko-ken-k3.ac.jp (H.M.); ishizuki@ko-ken-k3.ac.jp (C.I.); 2 Graduate School of Biomedical and Health Sciences, Hiroshima University, Hiroshima 734-8551, Japan; 3 Graduate School of Information Sciences, Hiroshima City University, Hiroshima 731-3194, Japan; fukuda@hiroshima-cu.ac.jp; 4 Health and Counseling Center, The University of Osaka, Toyonaka 560-0043, Japan; m.yasunaga@hacc.osaka-u.ac.jp

**Keywords:** fidgeting, auditory processing, pupil diameter, attention, arousal

## Abstract

**Highlights:**

**What are the main findings?**

- Both hand and leg fidgeting increased pupil diameter during auditory processing. Hand fidgeting produced the largest increase, indicating enhanced arousal or engagement rather than increased processing load.
- Despite these physiological changes, auditory task performance remained stable across all conditions. This suggests that mild fidgeting does not interfere with auditory processing in healthy young adults.

**What are the implications of the main findings?**

- Light fidgeting may serve as a simple, non-disruptive means of maintaining attention or preventing mind wandering during listening tasks.
- These results may inform educational or clinical approaches aimed at supporting attention regulation, particularly for individuals with attentional difficulties or auditory processing challenges. However, further evidence is needed.

**Abstract:**

**Background/Objectives:** People often engage in small, repetitive movements—or “fidgeting”—while listening. This behavior has traditionally been regarded as a sign of inattention. However, recent perspectives suggest that these movements may support engagement and arousal regulation. Yet, little is known about how different types of fidgeting affect the allocation of cognitive resources during auditory processing. This study examined whether hand and leg fidgeting influence pupil-linked arousal and auditory task performance. **Methods:** Young, healthy adults aged 18–26 years completed four auditory processing tasks while performing either hand fidgeting (manipulating a small fidget toy) or leg fidgeting (very light ergometer pedaling). A control group did not fidget. Pupil-linked arousal was assessed using changes in pupil diameter, and listening performance was evaluated across tasks of varying difficulty. **Results:** Both forms of fidgeting caused pupil dilation compared to the control group, particularly in the case of Hand Fidgeting during the listening task with speech in noise and the fast speech task. Despite these physiological changes, there were no measurable differences in auditory task performance across conditions. **Conclusions:** Fidgeting modulates pupil-linked arousal without impairing auditory processing in young, healthy adults. Hand fidgeting may help sustain engagement during demanding listening tasks. However, because the fidgeting was intentional and task performance approached ceiling or floor levels, these findings should be interpreted as preliminary. Future studies should examine whether fidgeting supports arousal maintenance or listening performance in individuals with attentional vulnerabilities or auditory processing difficulties.

## 1. Introduction

People often engage in small, repetitive movements, such as foot tapping or manipulating an object, during listening activities like lectures or phone conversations. These behaviors are broadly referred to as fidgeting and have traditionally been regarded as signs of restlessness or inattention [1,2]. However, accumulating evidence suggests that fidgeting may serve adaptive functions, such as sustaining attention or regulating arousal [1,3,4].

Listening is a fundamental cognitive skill that underlies language development, academic performance, and everyday communication [5,6,7]. Listening under challenging conditions requires sustained engagement and adaptive regulation of arousal [8,9,10]. In recent years, pupillometry has been widely used as a sensitive physiological index of listening-related processes, including arousal, attentional engagement, and listening effort [8,11,12,13,14,15,16]. Previous studies have demonstrated that pupil diameter increases as listening demands rise, such as during speech-in-noise perception or rapid speech [9,11,17,18], and follows an inverted U-shaped curve, decreasing when the load exceeds an individual’s cognitive resources [9,12,17,19]. Furthermore, it has been demonstrated that pupillary responses function as physiological markers of the ascending arousal system and are thought to be closely related to the activity of the locus coeruleus noradrenergic system (LC-NE system) [20,21]. These findings have contributed to a growing understanding of how auditory task demands are reflected in physiological responses.

However, most prior pupillometry studies in auditory processing have focused on stimulus-related factors, such as signal-to-noise ratio, speech clarity, or linguistic complexity, while participants remained physically still [12,22,23]. Consequently, little is known about how concurrent motor behaviors—commonly observed in everyday listening situations—interact with pupil-linked arousal during auditory processing. In particular, behaviors such as fidgeting, which involve small, repetitive movements and are frequently observed during sustained listening, have rarely been examined from a physiological perspective in auditory research. To our knowledge, no previous study has systematically compared different types of fidgeting-related movements while simultaneously measuring pupillary responses during auditory processing tasks.

Several mechanisms have been proposed to explain why fidgeting may improve attention. These mechanisms include increasing physiological arousal, providing minimal cognitive stimulation to suppress mind wandering, and offering brief mental relief to stabilize task engagement [1,3,24,25]. Previous studies have demonstrated that fidgeting can enhance performance in cognitive [3,26], memory [25], academic [24,27,28], and fine motor tasks [29] and that light physical activity in educational and workplace settings does not necessarily diminish productivity [24,25,26,27,30,31,32,33,34,35,36]. Nevertheless, the effects of fidgeting on listening performance, particularly under different auditory demands, remain largely unexplored. Studies of fidgeting from a physiological perspective are also limited. Light lower-limb movements, such as very mild pedaling, have been shown to increase pupil diameter and modulate arousal [20,37]. Meanwhile, object manipulation engages sensorimotor and associative cortical regions [38]. However, the specific processes linking fidgeting to auditory information processing have not yet been examined systematically.

Another challenge in this area of research is the heterogeneity of fidgeting methods, participant characteristics, and task types used in prior studies. This has hindered the development of a consistent methodological framework. Therefore, quantitatively evaluating the physiological effects of different fidgeting behaviors during auditory processing is an important next step. To address these issues, the present study aimed to clarify whether fidgeting behaviors affect pupil-related arousal during auditory processing using pupil diameter. Since auditory processing ability stabilizes in late adolescence [39,40], the study focused on young adult participants. We compared two common fidgeting behaviors: hand fidgeting with a small toy and light leg fidgeting with an ergometer. Based on previous findings [20,37], we hypothesized that both hand and leg fidgeting would modulate pupil-linked arousal during auditory processing, as reflected by changes in pupil diameter.

We further explored whether such physiological modulation would be accompanied by changes in auditory task performance, although no directional hypothesis regarding performance outcomes was specified. The primary objective of this study was to examine whether fidgeting modulates pupil-linked arousal during auditory processing in young adults. A secondary exploratory objective was to ascertain whether such physiological changes are accompanied by measurable alterations in auditory task performance.

## 2. Materials and Methods

This study was designed and reported in accordance with the APA Journal Article Reporting Standards (JARS) for experimental research.

### 2.1. Participants

Participants were students in their late teens to twenties attending medical vocational schools. The final sample comprised 33 participants (18–26 years old, mean age: 20.5 ± 1.4 years; 13 males and 20 females). A larger number of individuals were initially recruited to account for potential exclusions due to screening failures or missing physiological data. Before enrollment, all candidates received a detailed explanation of the study’s purpose and procedures and provided written informed consent. They were screened using a questionnaire to confirm adequate comprehension and hearing ability. Individuals with a history of hearing loss, recurrent acute otitis media, otitis media with effusion, or medical conditions contraindicating bicycle ergometer exercise were excluded. Audiological evaluation was conducted as a screening procedure to exclude participants with suspected peripheral hearing impairment. Pure-tone screening audiometry was performed at 1000 Hz and 4000 Hz at 30 dB HL in both ears, in accordance with the manufacturer’s instructions. This approach was adopted to ensure normal hearing sensitivity for speech perception tasks, rather than to provide a comprehensive clinical assessment of auditory processing abilities. Because the present study aimed to examine physiological modulation of pupil-linked arousal during auditory processing tasks in young, healthy adults, a full diagnostic audiological battery was not administered.

The required sample size was calculated using G*Power (ver. 3.1.9.2), based on Cohen’s conventions [41], with a two-tailed test, 5% significance level, 0.8 power, and 0.25 effect size, yielding a total sample size of 28 participants. A total of 39 individuals were recruited, and after excluding six participants with missing pupil diameter data, 33 participants were included in the final analysis. No participant discontinued the study after enrollment.

### 2.2. Ethical Considerations

All participants provided written informed consent prior to participation. The study protocol was approved by the Ethics Review Committee of Hiroshima University (Review No. E2022-0199; Approval Date: 2 December 2022).

### 2.3. Experimental Methods

#### 2.3.1. Experimental Environment

The experimental environment was illuminated at approximately 500 lx. The fixation point was set on a windowless wall to ensure that the fluorescent lights did not enter the participant’s field of view. Participants sat on an ergometer (fitness bike: NAGARABIKE, AFB4518, ALINCO. Osaka, Japan, hereafter referred to as the ergometer) with a desk positioned facing the wall. During the experiment, participants listened to audio sources through headphones (Razer Nari Essential, 2.4 GHz, Lazer. Irvine, CA, USA), while the pupil diameter was measured using an eye tracker (Tobii Pro Glasses 2, Ver. 1.0, Tobii AB (Tobii Technology). Stockholm, Sweden).

#### 2.3.2. Method of Presenting Auditory Tasks (Figure 1)

Auditory processing was assessed using the Auditory Processing Test (APT), a standardized test battery widely used in Japan for clinical and educational evaluation of auditory processing abilities [42,43]. The present study employed four subtests selected from the APT manual based on their relevance to everyday listening situations, particularly in educational environments. All tests were administered binaurally through headphones, and participants were instructed to maintain visual fixation throughout the tasks. Volume was adjusted to a level comfortable for each subject. The presentation order was “Fast-Speech Listening Task,” “Speech-in-Noise Task,” “Multiple-Speech Listening Task,” and “Auditory Attention Task.”

(A) Fast Speech Test

The fast speech test consisted of 20 short sentences, each comprising three words, presented at approximately twice the normal speaking rate (12–13 words/s). Sentences were presented binaurally with an inter-sentence interval of 7 s. Participants were instructed to verbally repeat each sentence immediately after presentation. Performance was scored as the percentage of correctly repeated sentences.

(B) Speech-in-Noise Test

The speech-in-noise test consisted of 36 two-syllable words presented binaurally under background noise. Stimuli were delivered at six signal-to-noise ratio (SNR) levels (+10, +5, 0, −5, −10, and −15 dB), with six words presented at each SNR level in randomized order. Participants were instructed to verbally repeat each word they perceived. Accuracy was calculated separately for each SNR condition.

(C) Multiple Speech Test

In the multiple speech test, three short sentences (2–3 words each) were presented simultaneously. One sentence was input to both ears, while the remaining two sentences were input to each ear separately. Participants were instructed to verbally repeat the sentence they perceived along the midline. Performance was evaluated as the percentage of correctly repeated target sentences.

(D) Auditory Attention Test

The auditory attention test involved sequential presentation of single-digit numbers (1–9) in random order, with inter-stimulus intervals of 1000, 2000, or 3000 ms. Participants were instructed to respond as quickly as possible by clicking a mouse only when the digit “9” was presented immediately after “1”. Reaction time and response accuracy were recorded. During the auditory attention test, participants responded by clicking a mouse placed on the desk directly in front of them. To maintain consistency across experimental conditions, participants were instructed not to keep their hands on the mouse during waiting periods in any condition.

In the hand fidgeting condition, participants manipulated the fidget toy with both hands during task performance. When a target stimulus occurred, they were instructed to temporarily release the toy and click the mouse without visually checking it. In the control and leg movement conditions, participants similarly kept their hands resting on the desk and clicked the mouse positioned in front of them without looking at it when a response was required. This procedure was adopted to minimize differences in motor preparation and visual input across conditions during the auditory attention task.

##### Order of Execution and Evaluation Strategy

The four APT subtests were administered in a fixed order (fast speech, speech-in-noise, multiple speech, auditory attention) within each experimental condition. This order was consistent across participants and conditions to minimize variability related to task switching. Short rest periods were provided between tasks as needed.

The four tests took approximately 19 min to complete. After completing all tasks, the participants rated the subjective difficulty of listening to each auditory stimulus using a visual analog scale (VAS) [44]. The difficulty level was evaluated using a 0–100 scale, with 0 indicating “very easy” and 100 indicating “very difficult”.

**Figure 1:**
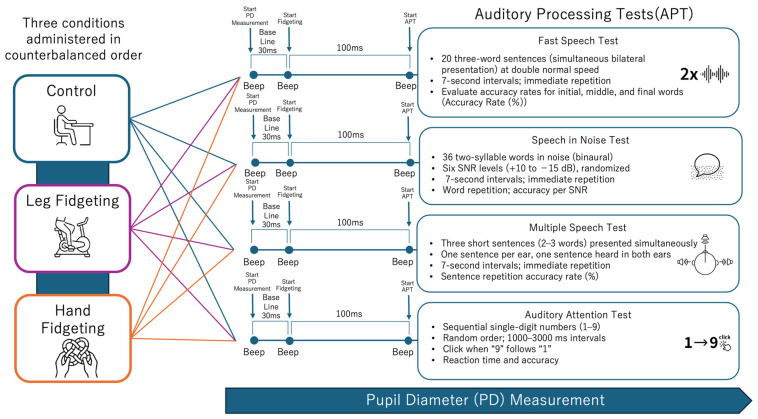
Schematic overview of the experimental protocol.

The figure illustrates the temporal structure of each experimental condition, including the baseline period, preparation period, and auditory processing test (APT) period. The sequence of the four APT subtests and the three experimental conditions (control, very light leg movement, and hand fidgeting) are shown. This schematic represents the experimental procedure only and does not depict task difficulty, performance outcomes, or pupil diameter data. This figure illustrates the experimental timeline and task structure. It does not represent task difficulty, performance, or pupil responses.

#### 2.3.3. Three Conditions for Fidgeting

To compare the effects of the presence and type of fidgeting on auditory processing, three conditions were established. All participants performed all three tasks. In each condition, the participants maintained fixation on a designated point on the wall to prevent gaze shift.

The first condition is the “control condition,” where APT is performed with both hands placed on the desk without pedaling on the ergometer. The second condition is “leg fidgeting condition”. This refers to very light leg pedaling and is one form of leg fidgeting operationally defined in this study. Ergometer driving began on the first beep, 10 s before the APT started, the APT began on the second beep, and driving continued until APT completion. Exercise intensity was set to 20–30% using the Karvonen method [45] and monitored using a heart rate monitor armband (XOSS_HRM_0036354) and a smartphone app (XOSS Ver. 3.11.4). The third condition was the “hand fidgeting condition,” where participants performed APT while operating a fidget toy (Tangle Classic Pomegranate, Tangle Creations, San Francisco, CA, USA) [28,46] with both hands. Participants began operating the fidget toy on the first beep, 10 s before the APT started, initiated the APT on the second beep, and continued fidgeting until completion.

The order of condition implementation was determined by counterbalancing. In particular, three types of condition-order sequences (3! = 6 possibilities) were created, and the participants were randomly assigned to these sequences. To eliminate the novel effects of the tools, the participants engaged in 3 min of ergometer cycling for leg fidgeting and free manipulation of the fidgeting toy for hand fidgeting before each experimental condition. A 5 min rest period in a seated position was provided between conditions to allow recovery of cognitive resources and auditory load [47,48].

### 2.4. Equipment

#### 2.4.1. Experimental System

The system consisted of a PC, an eye tracker, a data acquisition module (DAQ, UNI USB-6002, National Instruments, Austin, TX, USA), and the developed software. The software was built using C#.NET Framework 4.7 within the Visual Studio 2019 integrated development environment. NAudio is an audio library. The NET Framework was used to play WAV-formatted audio data. The .NET Framework API, which is compatible with the NI-DAQmx library, was used to control the DAQ. Audio output was confirmed to occur with a fixed delay of 10 ms, starting from the moment the synchronization signal output from the DAQ changed from low to high. This delay time was constant and equal to half of the eye tracker’s 20 ms sampling interval. Consequently, this delay did not affect the results of the pupil diameter analysis. The software enabled the presentation of auditory stimulus (APT) at arbitrarily set time intervals and generated TTL signals for synchronization with the eye tracker via the digital output port of the DAQ.

#### 2.4.2. APT

In Japan, the APTs developed by Hatta et al. [49] and Kaga et al. [42] are primarily used for auditory processing assessments. The APT by Kaga et al. was selected because it is considered useful not only in medical institutions but also in educational institutions, is designed for a wide range of individuals regardless of hearing impairment, and allows for the selection of test types and modification of test conditions based on the participant’s age and situation. The APT consists of the following tests: binaural separation hearing test, fast speech listening test, gap detection test, speech-in-noise test, binaural alternating listening test, auditory attention test, multiple speech listening test, and an additional test (a freely created test). From these, we selected four items particularly characteristic of the APD: fast speech test, speech-in-noise test, multiple speech test, and auditory attention test, and used them as auditory tasks.

The APT utilized in this study has been standardized and validated in prior research involving adolescents and young adults with normal hearing, as demonstrated in previous studies [42,43]. The normative performance ranges for each subtest have been reported in prior studies, demonstrating consistent performance across young adult populations without auditory difficulties. The normative data presented herein indicate that the selected tasks are suitable for assessing auditory processing abilities in this target age group.

#### 2.4.3. Eye Tracker

In recent years, diverse physiological indicators have been used alongside subjective measures in auditory science research [50,51,52]. Among these, the pupil diameter measurement stands out as a highly sensitive granular physiological indicator applicable to numerous research topics [11,12,13,14,15,16]. Pupil diameter is widely used as an excellent indicator for evaluating the central nervous system, capable of assessing arousal, cognitive processes, cognitive resource allocation, mind wandering, and more [8,11,12,13,14,15,16,22,53,54,55,56,57]. This technique has also been shown to reflect changes in cognitive load due to auditory stimuli and is particularly sensitive to the allocation of cognitive resources [12,57].

Pupil dilation generally indicates increased cognitive resource allocation and attention, while constriction indicates decreased allocation or mind wandering [11,12,22,23]. Moreover, pupil dilation reaches its maximum at moderate speech clarity levels, decreases at high clarity levels, and becomes slightly smaller at difficult levels, exhibiting an inverse U-shaped relationship [19,58,59]. These findings suggest that evaluating pupil diameter when fidgeting is introduced during auditory processing could serve as an effective neurological means of capturing the impact of fidgeting on cognitive resource allocation and mind wandering.

### 2.5. Data Analysis Methods

#### 2.5.1. Pupil Diameter Preprocessing

Pupil diameter data were recorded at a sampling rate of 50 Hz. Using the eye tracker analysis software Tobii Pro Lab 1.232, the data were exported to a CSV file, and the “Pupil diameter filtered” data were extracted. For preprocessing, a moving mean was applied to data from five points within a 100 ms rectangular window. The data were then divided into three intervals: the baseline interval (3 s before the first beep), preparation interval (10 s between the first and second beeps), and auditory stimulus interval (from the second beep onwards during the auditory task). A total of 12 datasets per participant (three conditions × four auditory tasks) were collected, resulting in 396 datasets.

**Figure 2:**
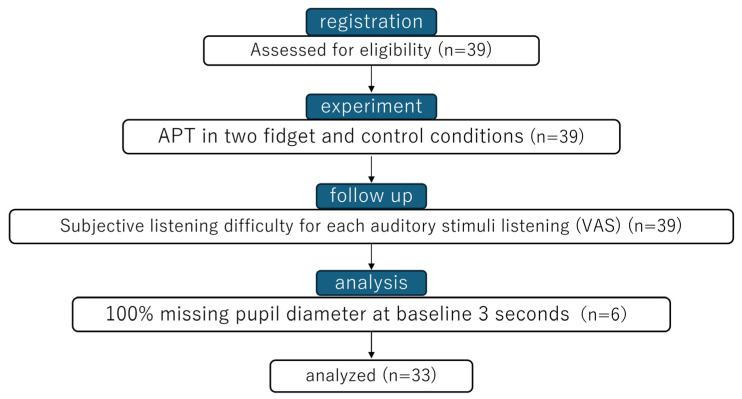
Participant Selection Process.

All pupil diameter data were excluded from the analysis if the baseline interval was missing, resulting in the exclusion of 6 participants (Figure 2). Preprocessing was customized for the Tobii Pro Glasses 2 data based on prior research [60]. Blink detection was performed using a velocity threshold for changes in pupil diameter. When the interval between blinks was 100 ms or longer, they were merged into a single long blink, and the data from the detected blink intervals were set to NaN. Preprocessed data were baseline-corrected by subtracting the baseline interval mean pupil diameter from the individual’s mean pupil diameter during APT, as previously described [61]. In this study, this value is referred to as the baseline-corrected pupil diameter.

#### 2.5.2. Analysis of Fidgeting Effects on Cognitive Resource Allocation

All data were tested for normality using the Shapiro–Wilk test. Because normality was rejected for some data, the Friedman test was used to compare the three conditions for a more robust analysis. Multiple comparisons, including Bonferroni correction, were performed when significant differences were observed. Additionally, for the speech-in-noise test within the APT, changes in the pupil diameter across different S/N ratios were compared using the same method.

#### 2.5.3. Analysis of Fidgeting Type Differences

The change in pupil diameter owing to hand and leg fidgeting was calculated by subtracting the baseline-corrected mean pupil diameter of the control group from the baseline-corrected mean pupil diameter of each fidgeting group. For the four APTs, pupil diameter changes in the leg and hand fidgeting conditions showed no significant deviations in the Q-Q plot. However, the Shapiro–Wilk test indicated deviations from normality for some data. For a more robust analysis, the two groups were compared using the Wilcoxon signed-rank test.

#### 2.5.4. Analysis of Fidgeting Effects on APT Performance

Normality was rejected for most items in the Shapiro–Wilk test for the correct answer rates of the four APTs; therefore, the Friedman test was used to compare the three conditions. The correct answer rate for each fidgeting condition was compared with the correct answer rate for the control condition by subtracting the rate for the control condition. This allowed for a comparison of the differences in the impact on correct answer rates between the leg-fidgeting and hand-fidgeting conditions. Since normality was rejected by the Shapiro–Wilk test, the Wilcoxon signed-rank test was used for the analysis.

All statistical analyses were performed using SPSS version 31.0 software (IBM Corp., Armonk, NY, USA).

## 3. Results

### 3.1. Participant Profile

No participants withdrew, except for six who were excluded during pupil diameter preprocessing; the final analysis included 33 participants (Figure 2). Table 1 shows the participant characteristics.

### 3.2. Pupil Diameter Changes During APT: Comparison Among Three Conditions

Table 2 summarizes baseline-corrected mean pupil diameter during APT across conditions and VAS-assessed subjective difficulty for each auditory task. The subjective difficulty (VAS) was the highest for fast speech, followed by speech-in-noise, multiple speech listening, and auditory attention. Significant differences were observed between the control and hand-fidgeting conditions for fast speech and speech-in-noise (fast speech, *p* = 0.002; speech-in-noise, *p* = 0.014).

For the S/N ratios in speech-in-noise tests, compared to the control condition, a significant difference was observed only at 0 dB in the leg fidgeting condition (*p* = 0.029, Bonferroni-adjusted). In the hand-fidgeting condition, significant differences were observed at all S/N ratios, except −5 dB and −15 dB (+10 dB: *p* = 0.014; +5 dB: *p* = 0.014; 0 dB: *p* = 0.003; −10 dB: *p* = 0.041, Bonferroni adjusted).

### 3.3. Comparison of Pupil Diameter Changes During APT Across Two Types of Fidgeting Conditions (Table 3)

To examine differences based on fidgeting type, we compared changes in baseline pupil diameter between the hand fidgeting and leg fidgeting conditions relative to the control condition during APT administration. Although no major deviations were observed in the Q-Q plot, the Shapiro–Wilk test indicated that some data were not normally distributed. Therefore, the Wilcoxon signed-rank test was used for the between-condition comparisons. The results showed significant differences between the two conditions for speech-in-noise and multiple speech.

Furthermore, analysis of the speech-in-noise test based on S/N ratio revealed significant differences at +10 dB, +5 dB, −10 dB, and −15 dB.

**Table 3:** Comparison of Pupil Diameter Changes (mm) During APT Implementation for Hand Fidgeting and Leg Fidgeting.

	Leg Fidgeting		Hand Fidgeting		*p*	r
	Mean	SD	Mean	SD		
Fast Speech	0.085	0.276	0.219	0.426	0.088	−0.30
Speech in Noise	0.111	0.291	0.282	0.457	0.049 *	−0.350
+10 dB	0.123	0.295	0.278	0.432	0.046 *	−0.058
+5 dB	0.109	0.295	0.284	0.456	0.046 *	−0.347
0 dB	0.141	0.314	0.299	0.469	0.067	−0.319
−5 dB	0.100	0.311	0.275	0.459	0.075	−0.310
−10 dB	0.083	0.298	0.253	0.455	0.046 *	−0.347
−15 dB	0.105	0.288	0.278	0.469	0.044 *	−0.366
Multiple Speech	0.027	0.321	0.161	0.438	0.046 *	−0.347
Auditory Attention	0.146	0.301	0.162	0.427	0.755	−0.054

The Wilcoxon signed-rank test with Bonferroni correction was used to compare the two conditions. Effect size (r) was calculated as r = Z/√n (n = 33) (r = 0.1 indicates a small effect size, r = 0.3 indicates a medium effect size, r = 0.5 indicates a large effect size). The *p*-values in the table are adjusted values, and * *p* < 0.05 was considered significant.

### 3.4. Comparison of APT Correct Answer Rates Across Three Conditions (Table 4)

The Friedman test comparing APT accuracy rates across the three conditions revealed no significant differences in any task.

**Table 4:** Comparison of APT Correct Answer Rates Across Three Conditions by Fidgeting Type.

		Reference Value (%)	Control (%)	Leg Fidgeting (%)	Hand Fidgeting (%)	*p*	W
Fast Speech	Initial words	96.7 (2.6)	79.24 (14.41)	80.00 (12.49)	79.24 (10.95)	0.568	0.017
Middle words		91.7 (5.2)	75.30 (17.75)	73.94 (19.14)	74.55 (16.21)	0.500	0.020
Final words		90.8 (4.9)	83.18 (18.42)	82.12 (17.37)	82.88 (14.98)	0.590	0.016
Speech in Noise		-	65.91 (4.75)	65.99 (5.21)	67.34 (5.16)	0.750	0.008
+10 dB		100.0 (0.0)	100.00 (0.00)	100.00 (0.00)	100.00 (0.00)	1.000	0
+5 dB		97.2 (6.9)	95.45 (8.48)	94.95 (9.61)	95.96 (9.20)	0.729	0.010
0 dB		94.3 (8.8)	94.44 (9.77)	94.95 (9.61)	94.95 (11.23)	0.814	0.006
−5 dB		66.8 (10.4)	68.18 (14.43)	69.70 (13.27)	70.20 (13.46)	0.907	0.003
−10 dB		14.0 (12.5)	21.72 (16.15)	19.70 (13.89)	21.21 (11.07)	0.532	0.020
−15 dB		2.8 (6.9)	17.17 (11.23)	16.67 (13.61)	17.17 (8.69)	0.912	0.003
Multiple Speech		94.2 (8.0)	79.85 (29.76)	76.97 (31.82)	77.88 (32.15)	0.771	0.008
Auditory Attention		95.8 (8.0)	98.18 (3.86)	96.36 (5.12)	96.82 (5.34)	0.120	0.064
Reaction Time (ms)		632.3 (77.3)	787.09 (264.23)	760.03 (228.53)	850.45 (230.89)	0.148	0.058

Values are presented as mean (SD). Comparisons across the three conditions (Control, Leg Fidgeting, and Hand Fidgeting) were performed using the Friedman test. Effect sizes are reported as Kendall’s W (W = χ^2^/[N (k − 1)]). No post hoc comparisons were conducted because no significant main effects were observed. The reference values are quoted from the manual [34] for individuals in their 20 s.

### 3.5. Comparison of APT Correct Answer Rates Across Two Types of Fidgeting Conditions (Table 5)

The Wilcoxon signed-rank test comparing the difference (change) between the leg fidgeting and hand fidgeting conditions relative to the control condition revealed a significant difference between the two groups only in the reaction time for the auditory attention test.

**Table 5:** Comparison of APT Correct Answer Rates Across Two Types of Fidgeting Conditions.

		Leg Fidgeting (%)		Hand Fidgeting (%)		*p*	r
		Mean	SD	Mean	SD		
Fast Speech	Initial words	0.76	11.22	0.00	9.13	0.594	0.09
Middle words		−1.36	18.23	−0.76	10.45	0.844	0.03
Final words		−1.06	14.50	−0.30	10.07	0.854	0.03
Speech in Noise		0.08	4.51	1.43	5.12	0.248	0.20
+10 dB		0.00	0.00	0.00	0.00	1.000	0.00
+5 dB		−0.51	11.23	0.51	8.69	0.420	0.14
0 dB		0.51	8.69	0.51	9.61	0.739	0.06
−5 dB		1.52	15.00	2.02	15.76	0.739	0.06
−10 dB		−2.02	15.76	−0.51	15.62	0.487	0.12
−15 dB		−0.51	13.29	0.00	10.05	0.827	0.04
Multiple Speech		−2.88	18.91	−1.97	16.92	0.806	0.04
Auditory Attention		−1.82	6.61	−1.36	6.99	0.676	0.07
Reaction Time		−27.06	155.71	63.36	230.38	0.004 *	0.50

The Wilcoxon signed-rank test with Bonferroni correction was used to compare the two conditions. Effect size (r) was calculated as r = Z/√n (n = 33) (r = 0.1 indicates a small effect size, r = 0.3 indicates a medium effect size, and r = 0.5 indicates a large effect size). The *p*-values in the table are adjusted values, and * *p* < 0.05 was considered significant.

## 4. Discussion

This study examined whether fidgeting influences pupil-related arousal regulation and auditory processing, and whether different types of fidgeting—hand versus foot movements—produce differential effects. Across all auditory processing tasks (APTs), both types of fidgeting were associated with pupil dilation during specific task conditions. Notably, in the fast speech and speech-in-noise tasks, hand fidgeting elicited greater pupil dilation than the other conditions. However, no changes in task performance were observed across tasks, and no overt behavioral interference was detected. This finding suggests that, under the present experimental conditions, the auditory tasks did not exceed the processing capacity of young healthy listeners. Accordingly, fidgeting did not result in observable behavioral interference, although this does not allow definitive conclusions regarding changes in cognitive resource allocation.

It is important to note that, while pupil dilation is widely interpreted as an index of arousal or engagement, it also possesses an aspect as a non-specific marker. Specifically, this may be indicative of increased cognitive effort, elevated attentional demands, or simply distraction. Consequently, the present findings do not permit a clear differentiation between arousal-modulating behaviors and effort-related processes.

### 4.1. Fidgeting, Arousal Modulation, and Performance Maintenance

Pupil dilation generally reflects attention, arousal, or increased cognitive engagement [8,53,57], particularly when task demands remain within an individual’s capacity [15,19,23,59,60,61]. In this study, pupil diameter continued to increase as the signal-to-noise (S/N) ratio decreased. This suggests that the auditory tasks did not exceed the cognitive limits of the listeners and that fidgeting did not create enough additional demands to cause dual-task interference [62,63].

Since listening is largely an automatic behavior for young adults [64], simple motor behaviors, such as fidgeting, may occur with minimal executive control. Under these circumstances, fidgeting may stabilize engagement, preventing mind wandering or attention drift, as reflected by greater pupil dilation without accompanying performance costs. This interpretation is consistent with prior findings that light movement can elevate arousal or cortical activation while leaving cognitive performance intact [65,66].

In the current study, auditory task performance remained stable across conditions. However, the absence of performance differences does not preclude the possibility that participants allocated additional cognitive effort when fidgeting, which may have been captured by pupillary responses. Consequently, the observation of pupil dilation during periods of fidgeting may be indicative not only of arousal regulation but also of compensatory cognitive effort.

It is also important to consider that the concurrent motor activities employed in this study were intentionally mild and controlled. Such activities may not have imposed sufficient dual task demands to disrupt auditory performance, while still requiring additional attention or executive resources. Under these circumstances, physiological indices such as pupil diameter may be more sensitive than behavioral measures, capturing subtle changes in processing that do not translate into observable performance differences.

### 4.2. Why Hand Fidgeting Produces Larger Pupil Dilation

#### 4.2.1. Sensory Engagement in Manual Movements

Although the hand fidgeting task required the intentional manipulation of a small toy, the movements were simple and repetitive and quickly became habitual. Such motor actions generally become semi-automatic and impose minimal cognitive load [67,68]. However, tactile stimulation and object manipulation provide richer sensory feedback than leg pedaling. This richer feedback may enhance LC–NE activation, thereby producing greater pupil dilation [69,70]. Since performance remained stable, these increases likely reflect enhanced arousal rather than additional cognitive effort.

#### 4.2.2. Motor and Arousal Networks

Hand movements activate extensive sensorimotor and somatosensory regions [70,71]. In contrast, light leg pedaling engages motor networks for rhythmic lower-limb control [37,52,72,73]. The locus coeruleus (LC) and its surrounding areas receive numerous presynaptic fibers from various sources, including the prefrontal cortex, central nucleus of the amygdala, lateral hypothalamus, bed nucleus of the stria terminalis, and dorsal raphe nucleus [69,74]. Both the hand and leg circuits provide afferent input to arousal-modulating systems, including the LC. It is plausible that hand fidgeting generates more varied or salient sensory input, leading to higher arousal levels. Importantly, this modulation appears to have occurred without drawing resources away from auditory processing.

#### 4.2.3. Variation Across Auditory Processing Tasks

The four APTs differed in complexity and cognitive demands [34]. Under the control condition, some tasks elicited minimal or decreasing pupil dilation, which is consistent with low engagement or mind wandering [56,75,76,77,78]. Fidgeting, especially hand fidgeting, counteracted this decline, suggesting that it plays a role in sustaining engagement, even during easier tasks. During difficult tasks, such as speech-in-noise and multiple-speech tasks, hand fidgeting increased pupil diameter at most signal-to-noise (S/N) levels. Generally, pupil diameter increases with task difficulty, but when task demands exceed an individual’s cognitive resources, pupil dilation typically declines, resulting in an inverted U-shaped relationship [19,58,59]. In the Speech in Noise Tasks of the present study, however, pupil diameter continued to increase with task difficulty and did not exhibit this classical pattern. Several factors may explain this finding.

First, even the most challenging auditory conditions may not have exceeded the participants’ available cognitive resources. The absence of performance decrements across tasks suggests that young healthy listeners retained sufficient capacity to manage the auditory demands, thereby preventing overload-related reductions in pupil dilation. Second, the auditory stimuli in the speech-in-noise task were brief—consisting of only one two-syllable word—which may have enabled participants to sustain intentional attention and listening effort even under low signal-to-noise ratio (SNR) conditions. This sustained effort may have contributed to comparable levels of pupil dilation across several difficult SNR levels. Third, concurrent fidgeting may have elevated or stabilized baseline arousal, leading to more sustained pupil dilation than would be observed under static listening conditions. Such motor activity may attenuate the emergence of the classical inverted U-shaped pattern typically reported in passive listening paradigms.

The APT results showed ceiling effects in tasks involving fast speech, speech-in-noise at +10 dB to 0 dB, multiple speech recognition, and auditory attention, while floor effects were observed in difficult speech-in-noise tasks at −10 dB and −15 dB, limiting the detection of subtle performance differences. These task characteristics contributed to consistent physiological but stable behavioral outcomes.

Taken together, these findings indicate that the U-shaped relationship between pupil dilation and listening difficulty is not preserved under conditions involving concurrent motor behavior. Rather than indexing task difficulty, pupil dilation in the present study appears to reflect context-dependent modulation of physiological state, underscoring the need for caution when generalizing pupillometric patterns across experimental paradigms.

### 4.3. Study Limitations

Several limitations require consideration:

(1) Fidgeting was intentional, not spontaneous:

The fidgeting behaviors implemented here involved voluntary toy manipulation or controlled pedaling. The leg condition involved controlled ergometer pedaling, which may partially reflect light exercise–related arousal rather than spontaneous fidgeting. Therefore, the physiological mechanisms underlying this condition may differ from those of naturalistic leg fidgeting. These differ from the natural, spontaneous fidgeting seen in daily life, which may involve distinct sensory or cognitive processes. Because fidgeting is inherently variable and difficult to quantify, future work should measure movement characteristics (e.g., frequency and intensity) and differentiate between spontaneous and intentional fidgeting.

(2) Ceiling and floor effects in APT performance:

Many auditory tasks yielded performance levels near the ceiling (easier conditions) or floor (most difficult signal-to-noise ratios [79]). This constrained our ability to detect subtle effects of fidgeting on behavioral performance.

Task parameters and procedures were determined based on standardized APT protocols and prior validation studies. However, no independent pilot study was conducted prior to the main experiment. Future research may benefit from pilot testing to optimize task sensitivity, particularly when applying similar paradigms to clinical populations.

(3) Ambiguity in interpreting pupil dilation:

A critical limitation of this study concerns the interpretation of pupil dilation. Because pupil diameter can reflect multiple overlapping processes, including arousal, cognitive effort, and distraction, the present data do not allow definitive attribution of the observed effects to arousal regulation alone. This ambiguity is particularly important given that the auditory tasks may have constituted only mild concurrent activity. Future studies incorporating converging measures, such as subjective effort ratings, EEG, or functional neuroimaging, will be necessary to disentangle these mechanisms.

(4) Limited generalizability:

Participants were young adults with normal hearing and normal auditory attention abilities. Therefore, the findings of this study may not fully generalize to other populations. Another limitation is that the audiological evaluation was limited to screening audiometry rather than a comprehensive diagnostic assessment. Although this approach was sufficient to exclude peripheral hearing impairment in the present healthy sample, future studies targeting clinical populations should incorporate full audiological batteries to more precisely characterize auditory processing abilities.

Thus, the effects observed in this study may differ in populations with hearing abnormalities identified through detailed audiological testing, vulnerabilities in attention function, hyperexcitability tendencies, or auditory processing disorder (APD).

### 4.4. Future Directions

Future research should examine whether fidgeting supports attentional regulation in individuals with APDs, ADHD, elderly individuals with MCI or those with fluctuating arousal levels. APD is also linked to developmental disorders [80]. In “hypo-arousal” conditions such as ADHD, reduced sustained firing in the LC-NE system lowers cortical arousal and impairs attention. In contrast, in “hyper-arousal” conditions such as anxiety disorders, increased sustained firing elevates cortical arousal, also disrupting attention [81]. Mild fidgeting may enhance engagement in hypo arousal states, though hyperarousal profiles may respond differently. Future research will first focus on young adults or children with APD, particularly those exhibiting attention deficits associated with reduced alertness. We will also examine the effects on tasks extending beyond brief stimuli, such as comprehension of longer passages and subsequent recall of their content. In addition to objective measures such as pupil diameter, subjective assessments of listening ease should also be evaluated. After that, it will also be important to evaluate fidgeting in naturalistic listening contexts, such as classrooms, lectures, and daily communication, to establish ecological validity. By doing so, distinguishing between spontaneous and intentional fidgeting and integrating neurophysiological measures will clarify how fidgeting interacts with auditory and attentional systems. Because this study involved only young adults with normal hearing and typical attentional abilities, caution is warranted when generalizing these findings to other populations. Understanding these mechanisms may inform the development of educational or clinical strategies that incorporate controlled motor activity to support listening.

## 5. Conclusions

The present findings indicate that both hand and leg fidgeting are associated with increased pupil diameter during auditory processing in young, healthy adults, without measurable changes in task performance. These results suggest that mild concurrent motor activity can modulate pupil-linked arousal under listening conditions that do not exceed processing capacity. However, because the study was exploratory and relied on pupil diameter as a physiological index, no definitive conclusions can be drawn regarding changes in cognitive resource allocation. Further studies using converging behavioral and neurophysiological measures are warranted.

## Figures and Tables

**Table 1:** Participant’s characteristics (n = 33).

Characteristics	All, n = 33
Age, mean (SD), years	20.52 (1.37)
Sex, n (%)	
Male	13 (39.39)
Female	20 (60.61)

**Table 2:** Comparison of Pupil Diameter (mm) After Baseline Correction Across Conditions (Control, Leg Fidgeting, and Hand Fidgeting).

	VAS (Subjective Difficulty of the Task) (SD)	Control (A)	Leg Fidgeting (B)	Hand Fidgeting (C)	Statistically Significant	*p*	r, η^2^
		Mean (SD)	Mean (SD)	Mean (SD)			
Fast Speech	64.3 (20.9)	0.02 (0.21)	0.10 (0.16)	0.24 (0.35)	A < C	0.002	0.36
Speech in Noise	61.6 (23.6)	−0.06 (0.41)	0.05 (0.15)	0.22 (0.37)	A < C	0.014	0.49
+10 dB		−0.12 (0.24)	0.00 (0.12)	0.16 (0.33)	A < C	0.014	0.49
+5 dB		−0.10 (0.22)	0.01 (0.16)	0.19 (0.37)	A < C	0.014	0.49
0 dB		−0.11 (0.26)	0.03 (0.16)	0.19 (0.39)	A < B, A < C	0.029, 0.003	0.45, 0.58
−5 dB		−0.06 (0.24)	0.04 (0.15)	0.21 (0.38)	n.s.	0.144	0.06
−10 dB		0.00 (0.23)	0.08 (0.17)	0.26 (0.36)	A < C	0.042	0.43
−15 dB		0.01 (0.22)	0.11 (0.17)	0.29 (0.38)	n.s.	0.078	0.08
Multiple Speech	45.2 (32.8)	−0.06 (0.23)	−0.03 (0.23)	0.11 (0.35)	n.s.	0.109	0.07
Auditory Attention	24.0 (1.80)	−0.19 (0.32)	−0.05 (0.23)	−0.03 (0.44)	n.s.	0.207	0.05

The table shows the results of the comparison of the mean baseline-corrected pupil diameter during the listening task across the three conditions using the Friedman test. Where significant differences are observed, the results of multiple comparisons are presented. Values represent the mean baseline-corrected pupil diameter. The *p*-values for multiple comparisons were adjusted using Bonferroni correction. Effect sizes (r) for significant differences detected by Friedman’s test were calculated as r = Z/√N (N = 33). Effect sizes (η^2^) for non-significant differences were calculated as χ^2^/N × (k − 1). Effect size: 0.10, Small; 0.30, Medium; 0.50, Large. n.s. indicates non-significant (*p* > 0.05).

## Data Availability

The data supporting the findings of this study are publicly available in the Zenodo repository (Repository Name: Zenodo; DOI: https://doi.org/10.5281/zenodo.17862981, accessed on 22 January 2026).

## Abbreviations

The following abbreviations are used in this manuscript:

APT	Auditory processing test
SNR	Signal-to-noise ratio
VAS	Visual analog scale
SMA	Supplementary motor area
LC-NE	Locus coeruleus–noradrenaline system
DAQ	Data acquisition module
